# Patient Characteristics and General Practitioners’ Advice to Stop Statins in Oldest-Old Patients: a Survey Study Across 30 Countries

**DOI:** 10.1007/s11606-018-4795-x

**Published:** 2019-01-16

**Authors:** Milly A. van der Ploeg, Sven Streit, Wilco P. Achterberg, Erna Beers, Arthur M. Bohnen, Robert A. Burman, Claire Collins, Fabio G. Franco, Biljana Gerasimovska-Kitanovska, Sandra Gintere, Raquel Gomez Bravo, Kathryn Hoffmann, Claudia Iftode, Sanda Kreitmayer Peštić, Tuomas H. Koskela, Donata Kurpas, Hubert Maisonneuve, Christan D. Mallen, Christoph Merlo, Yolanda Mueller, Christiane Muth, Ferdinando Petrazzuoli, Nicolas Rodondi, Thomas Rosemann, Martin Sattler, Tjard Schermer, Marija Petek Šter, Zuzana Švadlenková, Athina Tatsioni, Hans Thulesius, Victoria Tkachenko, Péter Torzsa, Rosy Tsopra, Canan Tuz, Bert Vaes, Rita P. A. Viegas, Shlomo Vinker, Katharine A. Wallis, Andreas Zeller, Jacobijn Gussekloo, Rosalinde K. E. Poortvliet

**Affiliations:** 1 Department of Public Health and Primary Care, Leiden University Medical Center, Hippocratespad 21, 2333 ZD Leiden, The Netherlands; 2 Institute of Primary Health Care (BIHAM), University of Bern, Bern, Switzerland; 3 Department of Family Medicine, Amsterdam UMC, location AMC, University of Amsterdam, Amsterdam, The Netherlands; 4 Department of General Practice, Erasmus Medical Center, Rotterdam, The Netherlands; 5 Vennesla Primary Health Care Centre, Bergen, Norway; 6 Irish College of General Practitioners, Dublin, Ireland; 7 Hospital Israelita Albert Einstein, São Paulo, Brazil; 8 Department of Nephrology and Department of Family Medicine, University Clinical Centre, University St. Cyril and Metodius, Skopje, Macedonia; 9 Faculty of Medicine, Department of Family Medicine, Riga Stradiņs University, Riga, Latvia; 10 Institute for Health and Behaviour, Research Unit INSIDE, University of Luxembourg, Luxembourg City, Luxembourg; 11 Department of General Practice and Family Medicine, Center for Public Health, Medical University of Vienna, Vienna, Austria; 12 Timis Society of Family Medicine, Sano Med West Private Clinic, Timisoara, Romania; 13 Department for Family Medicine, Health Center Tuzla, Medical School, University of Tuzla, Tuzla, Bosnia and Herzegovina; 14 Department of General Practice, University of Tampere, Tampere, Finland; 15 Family Medicine Department, Wroclaw Medical University, Wrocław, Poland; 16 Primary Care Unit, Faculty of Medicine, University of Geneva, Geneva, Switzerland; 17 Primary Care and Health Sciences, Keele University, Keele, Staffordshire ST5 5BG UK; 18 Institute of Primary and Community Care Lucerne (IHAM), Lucerne, Switzerland; 19 Department of Community Care and Ambulatory Medicine, Institute of Family Medicine Lausanne (IUMF), Lausanne, Switzerland; 20 Goethe-University, Institute of General Practice, Frankfurt/Main, Germany; 21 Center for Primary Health Care Research, Clinical Research Center, Lund University, Malmö, Sweden; 22 SNAMID (National Society of Medical Education in General Practice), Caserta, Italy; 23 Department of General Internal Medicine, Inselspital, Bern University Hospital, University of Bern, Bern, Switzerland; 24 Institute of Primary Care, University Hospital Zurich, University of Zurich, Zurich, Switzerland; 25 SSLMG, Societé Scientifique Luxembourgois en Medicine generale, Luxembourg City, Luxembourg; 26 Department of Primary and Community Care, Radboud University Medical Center, Nijmegen, The Netherlands; 27 Department of Family Medicine, Medical Faculty, University of Ljubljana, Ljubljana, Slovenia; 28 Ordinace Řepy, s.r.o., Prague, Prague, Czech Republic; 29 Research Unit for General Medicine and Primary Health Care, Faculty of Medicine, School of Health Sciences, University of Ioannina, Ioannina, Greece; 30 Department of Clinical Sciences, Section of Family Medicine, Lund University, Malmö, Sweden; 31 Department of Research and Development, Region Kronoberg, Sweden; 32 Primary Care, Region Kronoberg, Växjö, Sweden; 33 Department of Family Medicine, Institute of Family Medicine at Shupyk National Medical Academy of Postgraduate Education, Kiev, Ukraine; 34 Department of Family Medicine, Semmelweis University, Budapest, Hungary; 35 AP-HP, Assistance Publique des Hôpitaux de Paris, Université Paris 13, Paris, France; 36 Erzincan University Family Medicine Department, Erzincan, Turkey; 37 Department of Public Health and Primary Care, Universiteit Leuven (KU Leuven), Leuven, Belgium; 38 Department of Family Medicine, NOVA Medical School, Lisbon, Portugal; 39 Sackler Faculty of Medicine, Tel Aviv University, Tel Aviv, Israel; 40 Department of General Practice & Primary Health Care, School of Population Health Faculty of Medical and Health Sciences, The University of Auckland, Auckland, New Zealand; 41 Centre for Primary Health Care (uniham-bb), University of Basel, Basel, Switzerland

**Keywords:** hydroxymethylglutaryl-CoA reductase inhibitors, cardiovascular diseases, drug therapy, palliative care, general practitioners, clinical decision-making

## Abstract

**Background:**

Statins are widely used to prevent cardiovascular disease (CVD). With advancing age, the risks of statins might outweigh the potential benefits. It is unclear which factors influence general practitioners’ (GPs) advice to stop statins in oldest-old patients.

**Objective:**

To investigate the influence of a *history of CVD*, *statin-related side effects, frailty* and *short life expectancy*, on GPs’ advice to stop statins in oldest-old patients.

**Design:**

We invited GPs to participate in this case-based survey. GPs were presented with 8 case vignettes describing patients > 80 years using a statin, and asked whether they would advise stopping statin treatment.

**Main Measures:**

Cases varied in history of CVD, statin-related side effects and frailty, with and without shortened life expectancy (< 1 year) in the context of metastatic, non-curable cancer. Odds ratios adjusted for GP characteristics (OR_adj_) were calculated for GPs’ advice to stop.

**Key Results:**

Two thousand two hundred fifty GPs from 30 countries participated (median response rate 36%). Overall, GPs advised stopping statin treatment in 46% (95%CI 45–47) of the case vignettes; with shortened life expectancy, this proportion increased to 90% (95CI% 89–90). Advice to stop was more frequent in case vignettes without CVD compared to those with CVD (OR_adj_ 13.8, 95%CI 12.6–15.1), with side effects compared to without OR_adj_ 1.62 (95%CI 1.5–1.7) and with frailty (OR_adj_ 4.1, 95%CI 3.8–4.4) compared to without. Shortened life expectancy increased advice to stop (OR_adj_ 50.7, 95%CI 45.5–56.4) and was the strongest predictor for GP advice to stop, ranging across countries from 30% (95%CI 19–42) to 98% (95% CI 96–99).

**Conclusions:**

The absence of CVD, the presence of statin-related side effects, and frailty were all independently associated with GPs’ advice to stop statins in patients aged > 80 years. Overall, and within all countries, cancer-related short life expectancy was the strongest independent predictor of GPs’ advice to stop statins.

**Electronic supplementary material:**

The online version of this article (10.1007/s11606-018-4795-x) contains supplementary material, which is available to authorized users.

## INTRODUCTION

Cholesterol-lowering treatment with statins is an important part of cardiovascular risk management. Statins are frequently used in old age and continued to the end of life.^1, 2^ Statins can reduce the risk of cardiovascular events in old age.^3^ However, in case of life-limiting illness or multi-morbidity, the risk-to-benefit ratio of preventive statin treatment might favour stopping. Clarity and evidence about the point at which statin treatment is no longer beneficial is currently lacking.

General practitioners (GPs) are frequently confronted with the question ‘is statin treatment still appropriate for this older patient’. The lack of information about the risk-to-benefit ratio of preventive treatment for older patients makes it challenging for GPs to advise on statin therapy.^4, 5^ Guidelines generally do not include recommendations when to stop statin treatment (other than in the presence of adverse events).^6–9^ Statins have been identified as in need of evidence-based deprescribing guidelines.^10^ Recent research on GP decision-making on primary prevention of cardiovascular disease (CVD) suggests that, while some GPs follow guidelines regardless of patient age, other GPs take into consideration patient factors including comorbidities, frailty and estimated life expectancy.^4^ It is unclear how patient factors influence GPs’ advice on when to stop statin treatment in older patients.

In the present multi-national study, we investigated how patient characteristics (history of CVD, statin-related side effects, frailty and short life expectancy) influence GPs’ advice on stopping statins in patients over 80 years. We also investigated what other factors GPs consider relevant for stopping statin treatment.

## METHODS

### Setting

We conducted a survey of GPs (also called primary care provider) from 30 countries using 8 case vignettes. This study used the same approach for recruiting GP participants as the previous study conducted by this research group, that is using 29 GPs (National Coordinators) to coordinate recruitment and distribute the survey through a national GP network (for more details, see the “Procedure” section).^11^ Additionally, we recruited a National Coordinator for Belgium resulting in a total of 30 participating countries: Austria, Belgium, Bosnia and Herzegovina, Brazil, Czech Republic, Denmark, Finland, France, Germany, Greece, Hungary, Ireland, Israel, Italy, Latvia, Luxembourg, Macedonia, the Netherlands, New Zealand, Norway, Poland, Portugal, Romania, Slovenia, Spain, Sweden, Switzerland, Turkey, Ukraine and the UK.

### Participants

The National Coordinators distributed the survey across their educational or research networks of practicing GPs. We aimed for at least 20 participants per country. The number of GPs invited per network varied between 25 and 1100 GPs per country, with a median of 150.

Inclusion criteria for GPs were (1) the participant confirmed to be working as a GP and (2) the participant answered at least one question of the survey.

### Procedure

We used SurveyMonkey (www.surveymonkey.com, Palo Alto, CA, USA) to build the online survey (see Appendix 1). The National Coordinators translated the survey from English to their own language when necessary, making the survey available in 21 languages. In Israel and Finland, the National Coordinators distributed an English survey.

National coordinators distributed the survey by email. In Ukraine, where web access was limited, a paper survey was distributed. All National Coordinators were asked to send a first reminder after 2–3 weeks. Responses were collected anonymously (and labelled with a unique country code) between November 24, 2016, and April 11, 2017.

### Questionnaire

Appendix 1 represents the complete English survey.

#### GP Characteristics

We collected basic characteristics of participating GP and their practices including gender, years of experience, location of practice, and estimated proportion of patients aged over 80 years, and use of clinical practice guideline when treating patients aged over 80 years with statins (5-item Likert scale).

#### The Case Vignettes

The survey contained eight case vignettes. Each vignette described a patient aged over 80 years of unspecified gender consulting his or her GP for a routine follow-up. All vignettes described patients using a statin, with a low-density lipoprotein (LDL) level within the participants’ desired target range (not specified), and with no history of familial hypercholesterolemia.

To develop the case vignettes, we consulted 10 practising GPs from our network of regional practices and asked them to list their most important reasons for stopping statin treatment in patients aged over 80 years. We selected the four most frequent reasons for use in the survey vignettes: *absence of history of CVD*, *statin-related side effect*, *frailty*, and *short life expectancy.* For each vignette, participating GPs were asked if they would advise stopping statin treatment. Participating GPs were also asked if they would advise stopping statin treatment for each vignette if the patient additionally had a life expectancy of < 1 year due to a diagnosis of metastatic, non-curable cancer. Since predicting the prognosis of a patient can be difficult, the reason for limited life expectancy was added.

#### Characteristics of the Patients in the Case Vignette

##### History of CVD

Each case vignette included the medical history of the patient. It was explicitly stated whether CVD was present or not.

##### Statin-Related Side Effects

If present, the case vignette included the statement: ‘has complaints of myalgia, which is possibly a statin related side effect’. In the 4 case vignettes including statin-related side effects (case 2, 4, 6 and 8), GPs could choose to (a) stop statin treatment, (b) continue the same statin or (c) lower the current dose or switch to another type of statin. In further analyses, we dichotomized to ‘advise to stop statin’ (answer a) and ‘advise not to stop statin’ (answers b + c).

##### Frailty

Because frailty lacks a clear definition,^12^ we used the frailty definition by Fried et al. (2001),^13^ that is, unintentional weight loss, exhaustion, low level of activity, muscle weakness and slow gait speed. For each case vignette, we indicated one of the following statements: ‘You consider this patient to be frail’ or ‘You don’t consider this patient to be frail’.

To avoid order bias, the 8 cases were presented to participating GPs in a random order.

### Other Reasons for Stopping

We also asked participating GPs what factors they considered when stopping statin treatment in patients aged over 80 years, and which factor they considered most important.

### Statistical Analyses

We calculated proportions to describe categorised baseline data. For each of the case vignettes, we calculated proportions and 95%CIs for participants advising stopping statin treatment and the difference in proportions after adding short life expectancy to the case vignettes.

We calculated ORs and 95%CIs for advice to stop statin treatment using mixed-effect logistic regression models accounting for clustering within GPs and countries (adjusted in the model as random-effects factors). We performed crude and multivariable analyses, including the 4 patient characteristics, where we a priori chose to adjust for the GP characteristics (gender, years of experience [< 5; 5–20; > 20], location of practice [city, suburban, rural], estimated proportion of patients aged > 80 years [< 5; 5–20; > 20], and self-reported use of guidelines [mainly yes; neutral; mainly no]). We performed two sensitivity analyses of the multivariate model restricted to countries with a > 60% response rate (*n* = 7) to assess selection bias and restricted to countries where study team members were fluent in language to control for correct translations (*n* = 14).

To investigate international variation, we calculated the percentage of case vignettes with short life expectancy that GPs advised stopping statins and the ORs for the strongest independent predictor (short life expectancy) per country. Again mixed-effect logistic regression techniques were used including the 4 patient characteristics as fixed effects and GPs as random effects.

A two-sided *p* value below 0.05 was considered statistically significant.

Analyses were performed with SPSS version 22.0 (SPSS Inc., Chicago, Ill., USA) and with STATA 15.0 (StataCorp, College Station, TX, USA).

#### Data Availability

The datasets used and/or analysed during the current study are available from the corresponding author on reasonable request.

## RESULTS

### GP Characteristics

The survey was sent to a total of 10,048 GPs across 30 countries. The median response rate was 36% (range 7 to 93%) per country. Most responding GPs were from Switzerland (*n* = 497) and least from Greece (*n* = 15). Inclusion criteria were met by 2250 (95%) of the 2362 responders.

Table 1 presents the characteristics of the participating GPs: 54% were female, 50% practiced in a city, and 38% had > 20 years of experience working as a GP. Twenty-three percent of GPs estimated that more than 20% of their practice population were over 80 years.

**Table 1:** Characteristics of Participating GPs (*n* = 2250) from 30 Countries

GPs’ characteristics	*n* (%)
Female GP	1211 (54)
Practice location	
City	1134 (50)
Suburban	533 (24)
Rural	583 (26)
Experience as GP	
< 5 years	358 (16)
5–20 years	1024 (46)
> 20 years	865 (38)
Self-estimated prevalence of patients over 80 years at own practice	
≤ 20%	1697 (77)
> 20%	496 (23)
Treatment is based on (inter-)national guidelines	
Mainly yes	835 (43)
Neutral	498 (26)
Mainly no	611 (31)

### Case Vignettes

Table 2 shows the percentages of GPs advising stopping statin treatment for each case vignette with and without cancer-related short life expectancy. The number of completed case vignettes ranged between 1797 and 1806 with a mean of *n* = 1800.

**Table 2:** Proportion GPs Advising to Stop Statins in Older Patients with and Without Shortened Life Expectancy

Cases	Case characteristics			Proportion of GPs advising stopping statins (patients normal life expectancy)	Proportion of GPs advising stopping statins (patients with short life expectancy^‡^)
	CVD^†^	Side effects	Frailty	% (95%CI)	% (95%CI)
Overall				46 (45–47)	90 (89–91)
Case 1	–	–	–	50 (48–53)	91 (90–92)
Case 2	–	+	–	62 (59–64)	95 (93–96)
Case 3	–	–	+	82 (80–83)	95 (94–96)
Case 4	–	+	+	80 (78–82)	95 (95–97)
Case 5	+	–	–	5 (4–6)	77 (74–78)
Case 6	+	+	–	17 (16–19)	87 (85–89)
Case 7	+	–	+	35 (33–37)	86 (85–88)
Case 8	+	+	+	35 (33–37)	89 (87–90)

All case vignettes described one patient aged over 80 years of unspecified gender who consulted their GPs for a routine control. All patients already used statins, had an LDL level within the participants’ desired target range (not specified) and did not have a history of familial hypercholesterolemia.

†*CVD* cardiovascular disease

‡Life expectancy < 1 year

Overall, 46% (95%CI 45–47) of GPs advised stopping statin treatment in case vignettes without short life expectancy (range across cases 5 to 82%). In cases with short life expectancy, 90% of GPs advised stopping statins (95%CI 89–90) (range across case vignettes 77 to 95%).

In case vignette 1, describing a non-frail 82-year-old patient without CVD and without statin-related side effects, 50% (95%CI 48–53) of GPs advised stopping statin treatment; this increased to 91% GPs (95%CI 90–92) with added short life expectancy.

In case vignette 5, describing a patient with CVD but without statin-related side effects or frailty, only 5% of GPs (95%CI 4–6) advised stopping statin treatment. However, when short life expectancy was added, this increased to 76% of GPs advising stopping statin treatment (95%CI 74–78). Participants were most likely to advise stopping statin treatment in case vignettes 3 and 4.

Table 3 shows ORs from the logistic regression model testing the association of patient characteristics and GPs’ advice to stop statin treatment in patients aged over 80 years. Advice to stop statin treatment was more frequent in case vignettes without CVD compared to those with CVD (OR_adj_ 13.8; 95%CI 12.6–15.1). Advice to stop statin treatment was more frequent in case vignettes with statin-related side effects (OR_adj_ 1.6; 95%CI 1.5–1.7); or with frailty (OR_adj_ 4.1; 95%CI 3.8–4.4) compared to those without these characteristics. Addition of short life expectancy in the context of metastatic, non-curable cancer to the case vignettes increased advice of stopping (OR_adj_ 50.7; 95%CI 45.5–56.4) considerably. The two sensitivity analyses yielded similar results (data not shown). In case vignettes with statin-related side effects and without CVD (case 2 and 4), most GPs advised stopping statin treatment (62%; 95%CI 59–64 and 80%; 95%CI 78–82, respectively), but when statin-related side effects occurred in cases with CVD (case 6 and 8), most GPs advised continuing statin treatment in a lower dose or switching statin (74%; 95%CI 72–76 and 60%; 95%CI 57–62, respectively), rather than stopping (17%; 95%CI 16–19 and 35%; 95%CI 33–37, respectively).

**Table 3:** Association Between Patient Characteristics and GP Advice to Stop Statins in Patient > 80 Years

Characteristics	Univariate			Multivariate*		
	OR	95%CI	*P* value	OR_adj_	95%CI	*P* value
No cardiovascular disease	4.6	4.3 to 4.8	< 0.01	13.8	12.6–15.1	< 0.01
Side effects	1.3	1.2 to 1.4	< 0.01	1.6	1.5–1.7	< 0.01
Frailty	2.1	2.0 to 2.2	< 0.01	4.1	3.8–4.4	< 0.01
Short life expectancy	17.6	16.3 to 19.0	< 0.01	50.7	45.5–56.4	< 0.01

*Adjusted for GP characteristics (gender, experience, location, prevalence of oldest-old, guideline compliance) and patient characteristics (frailty, side effects, absence of cardiovascular disease and short life expectancy < 1 year). A mixed-effects model was used to account for multiple assessments of the 8 case vignettes per GP and per country

### Other Reasons to Stop Statin Treatment

**Figure 1:**
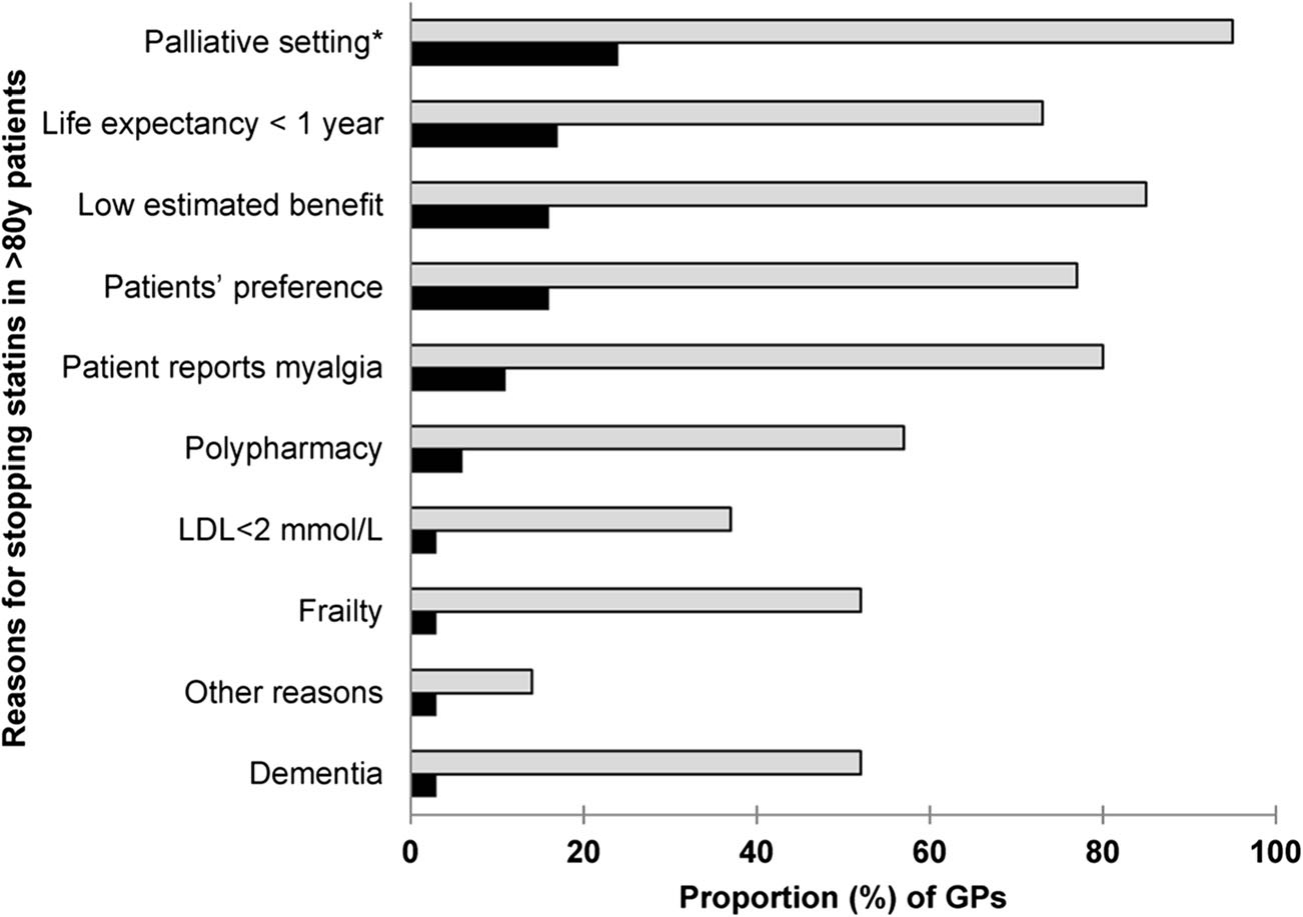
Reasons for GPs to stop statin treatment in patients over 80 years. The light grey coloured bars represent the proportion of GPs that consider this reason to advise to stop statins in patients over 80 years (multiple reasons could be selected). The black coloured bars represent the proportion of GPs that indicated this reason as the most important reason. Asterisk means life expectancy less than 3 months.

Figure 1 shows the factors that GPs said they considered when advising on stopping statin treatment in older patients. Participant GPs considered short life expectancy of unspecified cause the most important factor: 24% chose a life expectancy < 3 months and 17% a life expectancy < 1 year. Other factors GPs considered most important were patient preference (16%), and myalgia (11%). About half of GPs (52%) selected frailty as a reason to consider stopping statins, but only 3% considered frailty the most important reason.

Twelve percent of the GPs suggested other reasons for stopping including intolerance for the drug, hepatotoxicity and primary prevention.

### International Variation

The proportion of GPs advising stopping statin treatment in case vignettes with a short life expectancy in the context of metastatic non-curable cancer varied across countries, from 30% (95%CI 19–42) in Macedonia to 98% (95%CI 96–99) in Belgium. Cancer-related short life expectancy was the strongest independent predicator of GP advice to stop statin treatment.

See Appendix 2 for the results per country.

## DISCUSSION

In this international survey of more than 2200 GPs from 30 countries, we investigated how patient characteristics influence GP advice to stop statin treatment in patients 80 years and over. Overall, in 46% of the case vignettes, GPs advised stopping statin treatment (ranging across case vignettes from 5% to 82%). In case vignettes where life expectancy was < 1 year in the context of metastatic non-curable cancer, 90% of GPs advised stopping statin treatment (range across case vignettes 77 to 95%). The absence of CVD, the presence of statin-related side effects, and frailty were independently associated with GPs’ advice to stop statin treatment. There was considerable international variation in GP advice to stop statin treatment. Cancer-related short life expectancy was the strongest independent predictor of GP advice to stop statins, overall and across all countries.

### Context of the Results

When cases included short life expectancy (< 1 year), 9 out of 10 GPs advised stopping statin treatment. Our study confirms results from population-based studies showing that statin treatment is more likely to be discontinued in older people in the last year of life, independent of the need for primary or secondary prevention of CVD.^14, 15^ The importance of considering stopping preventive medication in older people with short life expectancy is increasingly recognised.^16^ We found that statin-related life expectancy was the main reason for GPs advising stopping statin treatment in patients aged over 80 years, consistent across countries. This study adds to the ongoing discussion about appropriate prescribing in older patients at the end of life reported in qualitative studies.^4, 5, 7^ Results of a recent trial suggest that stopping statin treatment in patients with short life expectancy (mean age 74.1 (SD 11.6) years) might be safe and improve quality of life.^17^ Although short life expectancy was the strongest independent predicator of GP advice to stop statin treatment in all countries, the proportion of GPs advising stopping statin treatment varied across countries. There are several possible explanations for this variation. A previous study showed that^18^ high CVD burden and low life expectancy at age 60 influences GPs decision to start antihypertensive treatment. These country-specific health characteristics could play a role in the decision to advice to stop statin treatment as well. In addition, perceptions of the patient population about statin therapy across the countries could play a role; these data were however not available for all countries.

We also investigated the influence of CVD on GP advice to stop statins in patients aged over 80 years. In cases with statin-related side effects, when CVD was present, most GPs advised lowering the dose or switching the type of statin; but when CVD was absent most GPs advised stopping the statin (Table 3). This suggests that GPs believe statins have a lower risk-to-benefit ratio for primary prevention than secondary prevention in patients aged over 80 years. However, current guidelines recommend treating patients with the maximum tolerated dose or using a different type of statin, irrespective of primary or secondary prevention.^8, 19^

While myalgia may be the main reason for statin discontinuation from a patient perspective, only 11% of GPs in our study chose side effects as the most important reason for stopping statins.^20^ The association between statin use and myalgia is still debated.^21, 22^

Consistent with the international literature, we found that GPs were more likely to advise stopping statin treatment when frailty was present.^4^

### Strengths and Limitations

This study has various strengths. First, this is the first international study addressing this problem with a case-based study. Second, we were able to include many respondents from many countries. Third, using case vignettes enabled us to investigate the influence of patient characteristics on standardised cases, which could not have been evaluated in a study based on real patients. Forth, case vignettes were presented in random order and clearly identified the factors to be considered when deciding whether to stop statins. Fifth, we completed sensitivity analyses for countries with a higher response rate (> 60%) and languages where the study team was fluent to control for correct translation returning similar results.

Our study has also some limitations. First, we used theoretical case vignettes. It can be argued that our results do not fully reflect daily practice and that social desirability bias and the premise of the survey could have influenced our results and lead to an overestimating of the willingness to stop statins. Although using statins in oldest-old with frailty and limited life expectancy is highly debated, we tried to use an as neutral as possible language when presenting the case vignettes. Further, we are confident that granting complete anonymity allowed GPs to freely express. Second, most GPs were part of national educational or research networks and might not be representative for all GPs. Third, we found some extremely high ORs (> 50) suggesting some findings should be interpreted with caution. Forth, we lost some respondents in the course of the survey. From the initial 2,250 GPs, about 1,800 (80%) responded to all questions of the survey. Given this proportion and by presenting the case vignettes in random order, we believe that risk of a selection bias is low. Fifth, we introduced the concept of limited life expectancy in the context of cancer; thus, our results might not be completely generalizable to the approach in patients with other conditions (e.g. severe heart disease). However, our respondents equally rated limited life expectancy (< 1 year) as the second important reason to consider stopping statins also in a general context not only limited to cancer (Fig. 1).

### Implications

The high proportion of GPs advising stopping statin treatment in patients with a cancer-related short life expectancy stresses the need for more research and clearer guidelines in this area. This dilemma—when to (discuss to) stop preventive medication—is relevant in any palliative care setting, independent of old age. However, in practice, it is difficult for doctors to accurately predict patients’ life expectancy.^23, 24^ It is important that the accuracy of such predictions are improved if they are to guide the discontinuation of preventive medicines.

A topic for further research and guideline development is the clarification of the risk-benefit profile of statins in older people when used for primary and for secondary prevention, given that GP advice to stop statins in the presence of statin-related side effects varied depending on whether the statin was being used for primary or secondary prevention.

## Electronic Supplementary Material

ESM 1 Complete English survey. Description of data: File 1 represents the complete English survey. (PDF 176 kb)

ESM 2 International variation. Description of data: File 2 shows the Odd ratios per country for GPs’ advice to stop statin treatment in patients aged over 80 years when life expectancy is less than 1 year. (PDF 128 kb)
