# Supplementary material for: Patient Characteristics and General Practitioners’ Advice to Stop Statins in Oldest-Old Patients: a Survey Study Across 30 Countries
Source: J Gen Intern Med. 2019 Jan 16;34(9):1751–7. doi: 10.1007/s11606-018-4795-x (PMC6711940; doi:10.1007/s11606-018-4795-x)
Supplement: Supplementary file 1 — Complete English survey. Description of data: File 1 represents the complete English survey. (PDF 176 kb) [file 11606_2018_4795_MOESM1_ESM.pdf]

Stopping cholesterol lowering medication in old age
Welcome

Dear colleague,

We welcome you to our international survey to understand treatment decisions of general practitioners (GPs) when they would advise their patient to stop cholesterol-lowering medication in oldest old patients (>80 years).

This study is conducted by the two Institutes of Primary Care in Leiden, the Netherlands, and Bern, Switzerland.

We estimate 5-10 minutes to complete this survey. All your answers are collected anonymously.

Kind regards,

Milly van der Ploeg, Sven Streit, Rosalinde Poortvliet and Jacobijn Gussekloo.

*1. Are you working as a general practitioner?

◯ Yes

◯ No (will result in exclusion from this study)

Stopping cholesterol lowering medication in old age
Basic characteristics

*** 2. What is your gender?**

◯ Female ◯ Male

*** 3. Where do you practice family medicine?**

◯ City

◯ Suburban

◯ Rural

*** 4. How many years have you been practicing as a general practitioner?**

◯ <5

◯ 5-10

◯ 11-15

◯ 16-20

◯ >20

Stopping cholesterol lowering medication in old age

Treating patients > 80 years

*** 5. What percentage of patients in your practice is 80 years or above?**

- ◯ <10
- ◯ 10-20
- ◯ 21-30
- ◯ >30

*** 6. When treating cholesterol, do you have a target LDL level for patients > 80 years of age?**

- ◯ Yes
- ◯ No

Stopping cholesterol lowering medication in old age

Target LDL level

***7. What is your target LDL level (treatment goal) in patients >80 years of age?**

***8. What is the <u>unit of measurement</u> of your target LDL?**

- ○ mmol/l
- ○ mg/dL
- ○ other, please specify

Stopping cholesterol lowering medication in old age
Guidelines

*** 9. My treatment goal for oldest-old is based on national or international guidelines?**

- ◯ yes
- ◯ mainly yes
- ◯ neutral
- ◯ mainly no
- ◯ no

*** 10. Please name the guideline you consider when treating hypercholesterolemia in patients >80 years?**

Stopping cholesterol lowering medication in old age
Cases

We will present you **8 different cases** and ask you to decide if you would advise your patient to stop cholesterol lowering medication (statin) in each case.

In each case we discuss an **oldest-old patient (>80 years) presenting at your GP office** for a routine visit. All have cholesterol lowering medication, have an LDL level within your target range and don't have a history of familial hypercholesterolemia.

We present only little information to you about these 8 cases but we do this on purpose to study a selected subset of patient characteristics. When we mention **frail**, this will be defined as patients with at least 2 of the following criteria: unintentional weight loss, exhaustion, low level of activity, muscle weakness and slow gait speed.

The **8 cases** will be presented in a **random order**.

Thank you for giving us your best response to each of these cases.

Stopping cholesterol lowering medication in old age
Case 1

82 year old patient, independent living with spouse

Findings

- no history of cardiovascular disease
- uses antihypertensive medication and a statin with LDL level within your target range
- has unintentional weight loss
- you consider this patient to be not frail

*** 11. Do you advise to stop cholesterol lowering treatment in this patient?**

◯ Yes
◯ No

*** 12. If this same patient was diagnosed with cancer with the presence of metastasis with no possibility of curation (life expectancy <1 year). Do you advise to stop cholesterol lowering treatment?**

◯ Yes
◯ No

Stopping cholesterol lowering medication in old age
Case 2

83 year old patient, independent living with spouse and some help in housekeeping

Findings
- no history of cardiovascular disease
- uses antihypertensive medication and a statin with LDL level within your target range
- has complaints of myalgia which is possibly a statin related side effect
- has slow gait speed
- you consider this patient to be not frail

*** 13. Do you advise to stop cholesterol lowering treatment in this patient?**

○ Yes
○ No, I would advise to continue
○ No, but I would advise to lower the current dosage or switch to another statin

*** 14. If this same patient was diagnosed with cancer with the presence of metastasis with no possibility of curation (life expectancy <1 year). Do you advise to stop cholesterol lowering treatment?**

○ Yes
○ No

Stopping cholesterol lowering medication in old age
Case 3

85 year old patient, living in a residence home with spouse, some help in housekeeping

Findings

- no history of cardiovascular disease
- uses analgesics, a diuretic and a statin with LDL level within your target range
- has exhaustion and slow gait speed
- you consider this patient to be frail

*** 15. Do you advise to stop cholesterol lowering treatment in this patient?**

◯ Yes
◯ No

*** 16. If this same patient was diagnosed with cancer with the presence of metastasis with no possibility of curation (life expectancy <1 year). Do you advise to stop cholesterol lowering treatment?**

◯ Yes
◯ No

Stopping cholesterol lowering medication in old age
Case 4

85 year old patient, living alone in a residence home

Findings
- no history of cardiovascular disease
- uses antihypertensive medication and a statin with LDL level within your target range
- has complaints of myalgia which is possibly a statin related side effect
- has low level of activity and slow gait speed
- you consider this patient to be frail

*** 17. Do you advise to stop cholesterol lowering treatment in this patient?**

○ Yes
○ No, I would advise to continue
○ No, but I would advise to lower the current dosage or switch to another statin

*** 18. If this same patient was diagnosed with cancer with the presence of metastasis with no possibility of curation (life expectancy <1 year). Do you advise to stop cholesterol lowering treatment?**

○ Yes
○ No

Stopping cholesterol lowering medication in old age
Case 5

80 year old patient, independent living with spouse

Findings
- patient suffered a myocardial infarction 2 years ago
- uses preventive cardiovascular medication including a statin with LDL level within your target range
- has low level of activity
- you consider this patient to be not frail

*** 19. Do you advise to stop cholesterol lowering treatment in this patient?**

◯ Yes
◯ No

*** 20. If this same patient was diagnosed with cancer with the presence of metastasis with no possibility of curation (life expectancy <1 year). Do you advise to stop cholesterol lowering treatment?**

◯ Yes
◯ No

Stopping cholesterol lowering medication in old age
Case 6

85 year old patient, independent living, some help in housekeeping

Findings

- suffered a myocardial infarction 3 years ago
- uses preventive cardiovascular medication including a statin with LDL level within your target range
- has complaints of myalgia which is possibly a statin related side effect
- has slow gait speed
- you consider this patient to be not frail

*** 21. Do you advise to stop cholesterol lowering treatment in this patient?**

◯ Yes
◯ No, I would advise to continue
◯ No, but I would advise to lower the current dosage or switch to another statin

*** 22. If this same patient was diagnosed with cancer with the presence of metastasis with no possibility of curation (life expectancy <1 year). Do you advise to stop cholesterol lowering treatment?**

◯ Yes
◯ No

Stopping cholesterol lowering medication in old age
Case 7

84 year old patient, independent living with spouse and some help in housekeeping

Findings
- suffered a stroke 3 years ago
- uses preventive cardiovascular medication including a statin with LDL level within your target range
- has muscle weakness and slow gait speed
- you consider this patient to be frail

*** 23. Do you advise to stop cholesterol lowering treatment in this patient?**

○ Yes
○ No

*** 24. If this same patient was diagnosed with cancer with the presence of metastasis with no possibility of curation (life expectancy <1 year). Do you advise to stop cholesterol lowering treatment?**

○ Yes
○ No

Stopping cholesterol lowering medication in old age
Case 8

81 year old patient, living in a residence home with spouse

Findings
- suffered a myocardial infarction 2 years ago
- uses preventive cardiovascular medication including a statin with LDL level within your target range
- has complaints of myalgia which is possibly a statin related side effect
- has unintentional weight loss and exhaustion
- you consider this patient to be frail

*** 25. Do you stop cholesterol lowering treatment in this patient?**

○ Yes
○ No, I would advise to continue
○ No, but I would advise to lower the current dosage or switch to another statin

*** 26. If this same patient was diagnosed with cancer with the presence of metastasis with no possibility of curation (life expectancy <1 year). Do you advise to stop cholesterol lowering treatment?**

○ Yes
○ No

Stopping cholesterol lowering medication in old age
Reasons for stopping

***27. In patients aged >80years which reasons would you have to stop cholesterol lowering treatment? (select all reasons which you have)**

○ LDL<2 mmol/L (75mg/dL)

○ Patients' preference

○ Frailty

○ Dementia

○ Polypharmacy

○ Patient reports myalgia

○ Life expectancy, less than one year

○ Palliative setting (life expectancy less than 3 months)

○ Low estimated benefit (e.g. low estimated rate of stroke reduction)

○ Others, please specify:

[ ]

***28. Which of the reasons do you consider to be the most important?**

[ ]

Stopping cholesterol lowering medication in old age
Starting reasons

***29. In patients aged >80years which reasons would you have to start cholesterol lowering treatment? (select all reasons which you have)**

◯ LDL>5 mmol/L (190 mg/dL)

◯ Patients' preference

◯ Vitality of the patient

◯ History of cardiovascular event

◯ History of diabetes

◯ Other cardiovascular risk factors (e.g. hypertension, smoking)

◯ High estimated benefit (e.g. high estimated rate of stroke reduction)

◯ I do not start cholesterol lowering medication in patients aged >80 years of age

◯ Others, please specify:

____

***30. Which of the reasons do you consider to be the most important?**

____

Stopping cholesterol lowering medication in old age

Final question

***31. Please give us feedback or comments to this survey.**

***32. Thank you for your participation! If you wish to be informed about the results, please type your email address.**

Kind regards,

Milly van der Ploeg, Sven Streit, Rosalinde Poortvliet and Jacobijn Gussekloo.
