# Supplementary material for: Patient Characteristics and General Practitioners’ Advice to Stop Statins in Oldest-Old Patients: a Survey Study Across 30 Countries
Source: J Gen Intern Med. 2019 Jan 16;34(9):1751–7. doi: 10.1007/s11606-018-4795-x (PMC6711940; doi:10.1007/s11606-018-4795-x)
Supplement: Supplementary file 2 — International variation. Description of data: File 2 shows the Odd ratios per country for GPs’ advice to stop statin treatment in patients aged over 80 years when life expectancy is less than 1 year. (PDF 128 kb) [file 11606_2018_4795_MOESM2_ESM.pdf]

Country (n)	% cases advised to stop (95%CI)	Odds Ratio (95%CI)
Luxembourg (16)	85 (79-89)	1252 (95 -16461)
Norway (19)	97 (93-100)	1049 (114-9692)
Belgium (73)	98 (96-99)	395 (165-943)
Portugal (46)	83 (77-88)	269 (95-764)
New Zealand (60)	94 (90-96)	263 (104-663)
Ireland (35)	91 (85-95)	233 (81-671)
Netherlands (289)	95 (85-95)	231 (159-336)
Austria (23)	83 (74-91)	196 (48-806)
Israel (107)	98 (85-92)	102 (60-176)
Romania (27)	78 (70-84)	100 (38-261)
Switzerland (469)	88 (86-89	86 (67-111)
Slovenia (27)	81 (70-90)	84 (23-308)
Denmark (18	90 (80-96)	69 (20-238)
Germany (63)	85 (79-89)	64 (33-124)
Italy (37)	80 (74-86)	59 (27-129)
United Kingdom (19)	77 (67-84)	57 (21-156)
Sweden (33)	78 (70-85)	53 (21-131)
Finland (32)	83 (77-89)	47 (23-96)
Hungary (85)	70 (66-75)	35 (23-55)
Czech Republic (13)	71 (58-81)	21 (8-58)
Brazil (43)	67 (60-74)	20 (11-36)
Greece (13)	69 (56-80)	19 (7-51)
Spain (32)	77 (67-85)	19 (9-38)
Poland (35)	45 (38-53)	18 (9-35)
Latvia (86)	63 (58-68)	18 (12-26)
France (18)	79 (70-89)	17 (7-40)
Turkey (39)	71 (64-78)	16 (9-27)
Bosnia Herzegovina (23)	76 (64-86)	9 (4-18)
Ukraine (65)	48 (43-54)	6 (4-9)
Macedonia (14)	30 (19-42)	3 (1-7)
**Overall (1859)**	**90 (89-90)**	**51 (46-57)**

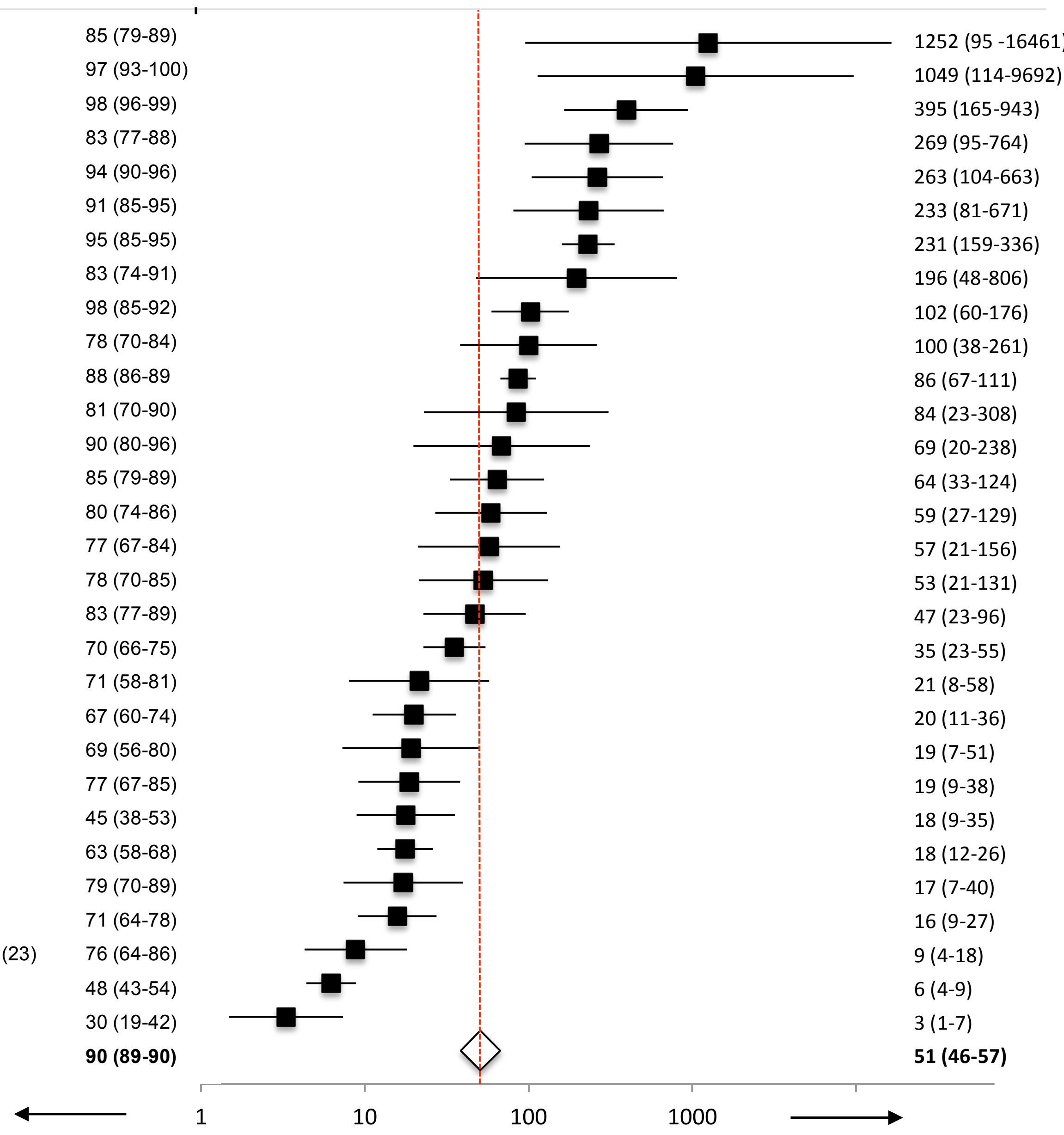

Favour not stopping treatment

Favour stopping treatment

Figure 1. Odd ratios[1] per country for GPs' advice to stop statin treatment in patients aged > 80 years when life expectancy is < 1 year.

[1]Adjusted for patient characteristics (frailty, side effects and absence of cardiovascular disease) A mixed-effects model was used to account for multiple assessments of the 8 case per GP.
